# Titan as the Abode of Life

**DOI:** 10.3390/life6010008

**Published:** 2016-02-03

**Authors:** Christopher P. McKay

**Affiliations:** Space Science Division, NASA Ames Research Center, Moffett Field, CA 94035, USA; Chris.McKay@nasa.gov; Tel.: +1-650-604-6864

**Keywords:** Titan, life, methane, second genesis

## Abstract

Titan is the only world we know, other than Earth, that has a liquid on its surface. It also has a thick atmosphere composed of nitrogen and methane with a thick organic haze. There are lakes, rain, and clouds of methane and ethane. Here, we address the question of carbon-based life living in Titan liquids. Photochemically produced organics, particularly acetylene, in Titan’s atmosphere could be a source of biological energy when reacted with atmospheric hydrogen. Light levels on the surface of Titan are more than adequate for photosynthesis, but the biochemical limitations due to the few elements available in the environment may lead only to simple ecosystems that only consume atmospheric nutrients. Life on Titan may make use of the trace metals and other inorganic elements produced by meteorites as they ablate in its atmosphere. It is conceivable that H_2_O molecules on Titan could be used in a biochemistry that is rooted in hydrogen bonds in a way that metals are used in enzymes by life on Earth. Previous theoretical work has shown possible membrane structures, azotosomes, in Titan liquids, azotosomes, composed of small organic nitrogen compounds, such as acrylonitrile. The search for a plausible information molecule for life in Titan liquids remains an open research topic—polyethers have been considered and shown to be insoluble at Titan temperatures. Possible search strategies for life on Titan include looking for unusual concentrations of certain molecules reflecting biological selection. Homochirality is a special and powerful example of such biology selection. Environmentally, a depletion of hydrogen in the lower atmosphere may be a sign of metabolism. A discovery of life in liquid methane and ethane would be our first compelling indication that the universe is full of diverse and wondrous life forms.

## 1. Introduction

The search for life on other worlds is often characterized by the strategy of “follow the water”. Water provides a convenient shorthand for the range of attributes that support life on Earth, including environmental processes associated with cycling of water, biochemistry of carbon in the medium of water, and the ecological systems that water-based life creates in water-cycling environments. Many of these attributes of the Earth, as an abode for life, are taken as given once the presence of liquid water is established. Thus, we can motivate a search for life on early Mars simply by the clear indication of liquid water on its surface, secure in the background knowledge of the environmental, biochemical, and ecological correlates of the widespread presence of liquid water.

It is important to keep in mind that one cannot really separate chemistry from environment. The role of water as a solvent for life on Earth depends on its chemical reactivity and on its physical properties, and the two are closely related. Thus, considering other liquids requires addressing the needs life has for chemical functionality, both in terms of physicochemical and reaction-chemistry-related requirements.

If we turn to the possibility of life based on another liquid, each of these background assumptions must be reexamined. The only other world we know that has a liquid on its surface is Titan, where we find liquids of methane and ethane. Titan is the largest moon of the planet Saturn but is small compared to Earth, with a surface gravity 1/7 that of Earth. Its surface atmospheric pressure is about 1.5 times that of Earth, and the surface temperature is 95 K. The atmosphere is dominated by N_2_ (95%) with CH_4_ (5%) and H_2_ (0.1%) and various trace organic compounds. The lower atmosphere has an active hydrological cycle of liquid methane, including convective clouds and rain, both of which vary with the seasons. Titan is locked in a synchronous orbit around Saturn with a period of 16 days (the cycle of light and dark). The tilt of Saturn’s spin axis to its orbit plane is ~27°, resulting in seasonal changes in the position of the sun in Titan’s sky over the ~30-year period of Saturn’s orbit. The lower atmosphere of Titan is too thick to respond to the 16-day light-dark cycle but does respond on the 30-year seasonal timescale. Thick convective clouds congregate in the summer polar region.

Photochemical reactions beginning with the dissociation of the atmospheric N_2_ and CH_4_ create an array of organic molecules in Titan’s atmosphere and produce a solid organic haze in the upper atmosphere that obscures the lower atmosphere and surface. The organic haze particles eventually settle on the surface. A major product of the photochemistry is ethane, which accumulates on the surface and mixes with liquid methane. There are large lakes of methane and ethane on Titan, ranging in size up to ~1000 km across. Observations of the Huygens lander indicated moisture (methane and ethane) in the ground at the landing site. It appears that liquid methane and ethane are widespread and actively cycled on Titan. Is a “follow the methane” strategy plausible?

In a seminal paper that considered the range of possible chemistries for life, Benner *et al.* [1] (see also Bains [2]) speculated that hydrocarbons that are naturally liquid on Titan could be a solvent for life. Benner *et al.* [1] noted that the organic reactivity in hydrocarbon solvents is no less versatile than in water, and indeed the ability to exclude water is an important aspect of many catalytic sites. Perhaps there could be life in Titan’s liquids.

The purpose of this paper is to develop an approach to characterizing Titan as a possible abode of life by examining the possibilities of physical environments created by the presence and cycling of Titan’s liquid, the biochemistry of carbon in that liquid, and the ecological systems that life might develop in that liquid. The case of water and life on Earth is the only guide we have for Titan as an abode of life, and so we must try to separate those features of habitability on Earth that are specific to Earth from those features that might be generalized to other liquids on other worlds.

## 2. The Global Environment

On Earth, life is connected to the global environment. Habitability is determined by the physico-chemical interactions of the fluids (water on Earth) in a specific environment, and by the solids present in that environment. Through physical processes such as sunlight and volcanism and their interaction with cycles of water, important physical conditions for life are established on the Earth. These include: (1) sources of chemical or light energy suitable for life; (2) nutrients; (3) liquid habitats; and (4) transport cycles of liquid moving nutrients and wastes. Do these also exist on Titan?

### 2.1. Sources of Chemical or Light Energy Suitable for Life

The area related to the habitability of Titan that has received the most detailed attention is the possibility of chemical energy sources. Schulze-Makuch and Grinspoon [3] and McKay and Smith [4] noted that the photochemically produced organics in Titan’s atmosphere would produce energy if reacted with atmospheric H_2_, and that this could be a source of biological energy. McKay and Smith [4] quantified the energy released from such reactions (Table 1). Hydrogenation of C_2_H_2_ provided a particularly energetic reaction, with 334 kJ per mole of C_2_H_2_ consumed. This can be compared to the minimum energy required to power methanogen growth on Earth of ~40 kJ mole^−1^, determined by Kral *et al.* [5], or the energy from the reaction of O_2_ with CH_4_, which produces ~900 kJ mole^−1^. The reactions listed in Table 1 are exothermic but kinetically inhibited at Titan temperatures. This is ideal for biology. For example, the reaction of O_2_ with CH_4_ is kinetically inhibited at Earth temperatures, but methane-oxidizing microorganisms present in the environment catalyze the reaction and thus derive energy from it.

**Table 1:** Free energy released by hydrogenation of hydrocarbons on Titan, per mole of hydrocarbon consumed [4].

Reaction	Energy Released (kJ mole^−1^)
C_2_H_2_ + 3H_2_ = 2CH_4_	334
C_2_H_6_ + H_2_ = 2CH_4_	57
R–CH_2_ + H_2_ = R + CH_4_	54

Given that redox couples (e.g., C_2_H_2_, 3H_2_) are produced in the atmosphere, and that these small molecules will be widespread on Titan and are readily soluble in liquid ethane and methane [6], there may not be any need for photosynthesis on Titan. However, nonetheless, it is interesting to consider the availability of sunlight on Titan’s surface. Models of light penetration in Titan’s atmosphere [7], and direct measurements by the Huygens Probe [8] provide a good understanding of the availability of sunlight in the atmosphere and at the surface. Because of Titan’s distance from the sun (10 AU) and the haze in the atmosphere, the maximum level of sunlight on the surface of Titan is about 0.1% that of the overhead sun on Earth’s surface [9]. Despite the haze, the distribution of solar flux with wavelength roughly follows the solar spectrum, with the peak flux occurring at about 0.6 um (Figure 11 of [8]). This low-light level is more than adequate for photosynthesis on Earth—which process can utilize light levels as low as 10^−6^ of the Earth noonday solar flux [10,11]. Thus, photosynthesis should be possible on Titan with pigments that would not be dissimilar to those used on Earth. Even with attenuation by the atmospheric haze and the large distance from the sun, the energy in sunlight reaching the surface of Titan is orders of magnitude larger than the chemical energy in hydrocarbons descending from the upper atmosphere. As on Earth, the biosphere on Titan would be most productive if it were to use solar energy directly. On Earth, photosynthesis is used to produce organic material primarily from CO_2_ and H_2_O. On Titan, presumably, photosynthesis should produce organic material from CH_4,_ and H_2_ would be a byproduct—the reverse of the reactions listed in Table 1. This is similar to the suggestion for photosynthesis in hydrogen-dominated atmospheres [12].

### 2.2. Nutrients

Organic compounds are common on Titan, including N containing organics. Thus, C, H, and N are available in multiple compounds. The surface contains H_2_O as ice but no other compounds with O atoms are common at the surface. Thus, life on Titan may have a restricted set of elements as nutrients, compared to the wide variety available and used by life on Earth. Bains and Seager [13] have pointed out that a small set of elements may have implications for the complexity of possible biochemistry. Small hydrocarbons and N_2_ are readily soluble in liquid methane [6], and some N and O containing molecules may be present in the liquid as well, as listed in Table 2.

**Table 2:** Molecules with O or N in liquid CH_4_ and C_2_H_6_ [6].

Compound	Solubility, Mole Fraction	
	Methane	Ethane
H_2_O	10^−22^–10^−18^	10^−18^–10^−15^
CO_2_	10^−5^–10^−3^	10^−5^–10^−3^
HCN	10^−12^–10^−10^	10^−10^–10^−8^
C_2_N_2_	10^−8^–10^−7^	10^−7^–10^−6^
CH_3_ CN: acetonitrile	10^−12^–10^−10^	10^−10^–10^−8^
C_2_H_3_CN: acrylonitrile	10^−12^–10^−10^	10^−10^–10^−8^

Schulze-Makuch *et al.* [14] suggested that a critical problem for life on Titan would be access to inorganic elements, such as Fe, Cu, Mn, Zn, Ni, S, Ca, Na, and K, *etc.*, which life on Earth can access through their solubility in water. These inorganic elements are used, often in trace levels, in various life functions. Of particular interest is the use of metals in the active sites of enzymes.

We can consider two approaches for nutrients to support life on Titan: (1) the conservative use of elements that are hard to access; and (2) the use of H_2_O to fill some of the roles of the inorganic elements.

The biology and environmental cycling of phosphorous on Earth provides an example of the conservative use of an element that is not readily available in the environment and is not naturally cycled by the physical transport systems in the environment. A similar conservative use may be needed on Titan for inorganic elements. A possible source of inorganic elements is the influx of meteorites and comets in the upper atmosphere. This source is known to be responsible for CO, CO_2_, and H_2_O in the atmosphere [15]. Thus, a small but possibly biologically useful flux of inorganic elements is descending from the upper atmosphere with the haze and may be adequate for life forms that carefully recycle and reuse these elements.

The alternative to a conservative use of trace inorganic elements for biology on Titan is to eliminate the need for these elements entirely. If life on Titan does not employ photosynthesis, and does not need to fix nitrogen from N_2_ (N is available in organic compounds produced photochemically), then two of the major needs for metal-based catalysis used by Earth life are removed. Furthermore, it is conceivable that H_2_O molecules on Titan could be used in a way that metals are used in enzymes by life on Earth. Because of the low temperatures on Titan, hydrogen bonds provide enough binding strength to form useful structures. Benner *et al.* [1] suggested that hydrogen bonding is difficult to use in the assembly of supramolecular structures in water, and they speculated that life in Titan liquids would be able to use hydrogen bonding more, and that these bonds would have the strength appropriate for the low temperature. The hydrogen bond (5 to 30 kJ mole^−1^) is stronger than a van der Waals interaction, but weaker than covalent (~300 kJ mole^−1^) or ionic bonds (20–30 kJ mole^−1^). Thermal energy, RT, at the Titan temperature of 95 K equals about 1 kJ mole^−1^. As a polar molecule (one of the few in the Titan environment), H_2_O is particularly good at affecting hydrogen bonds. Individual H_2_O molecules or small clusters held in hydrocarbon cages could play the catalytic role for hydrogen-bonded structures that metals do for redox reactions in Earth biochemistry.

### 2.3. Liquid Habitats

**Figure 1:**
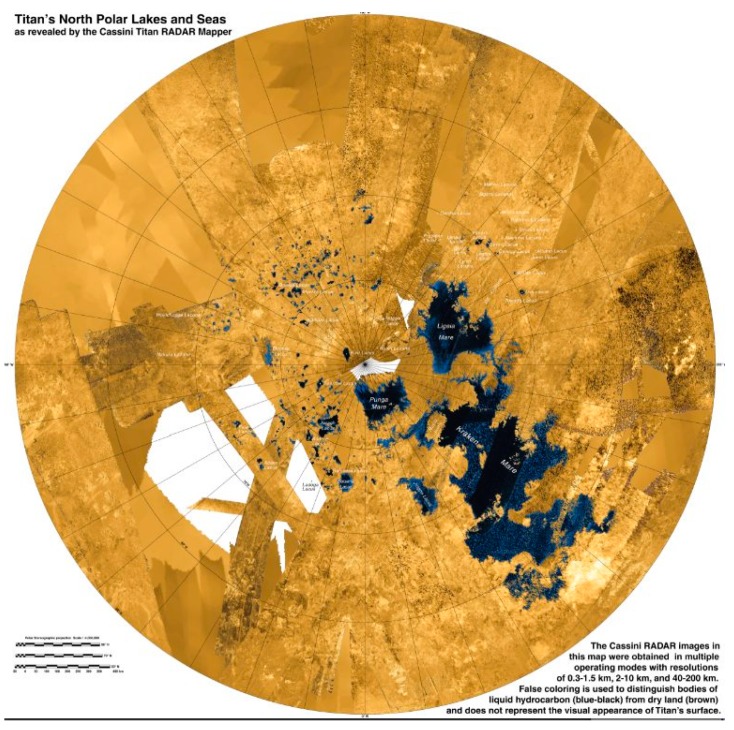
Lakes on Titan. False color map of the Northern Hemisphere down to 50°N shows Titan’s lakes in blue and areas that are not lakes in brown. The map is based on data from the radar on the Cassini spacecraft, taken during flybys between 2004 and 2013. Kraken Mare, the largest known lake on Titan is shown to the right and below the pole in this image. Above and right of the pole is Ligeia Mare, the second largest known lake. Image from NASA.

On Earth, life is widespread because habitable liquid water is widespread. Even in the driest desert on Earth, the Atacama Desert of Chile, there is occasional liquid water. Liquid on Titan is also widespread. There are large lakes in the northern hemisphere (Figure 1) and at least one large lake in the southern hemisphere [16]. Observations from the Huygens Probe indicated that the soil at the equatorial landing site was moist with methane and ethane [17,18] and, by extension with orbital spectral data, most of the low latitude soils are moist [19]. There may be low latitude lakes [20,21].

**Figure 2:**
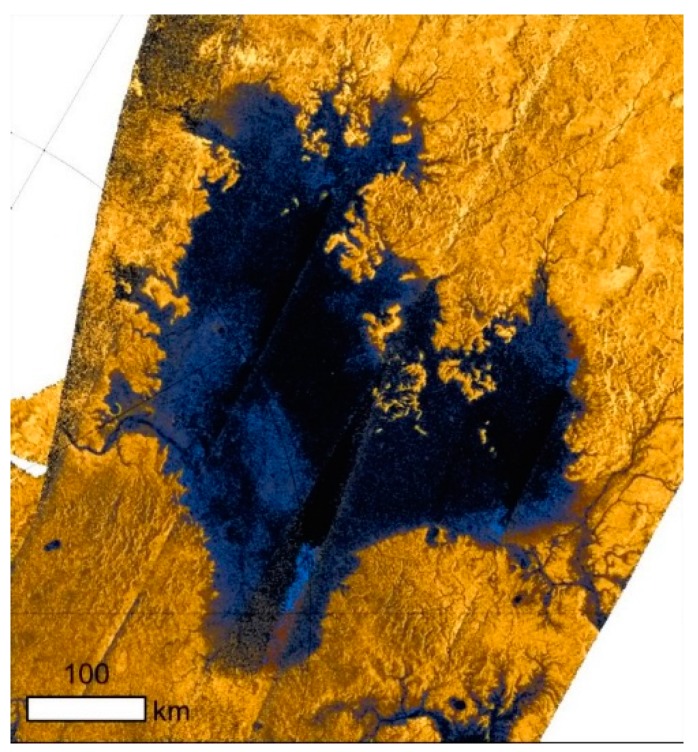
Close-up radar image of Ligeia Mare, showing coastline features and rivers. The lake is ~400 km across. Image from ESA based on Cassini Data.

The nature of the lakes, the processes that fill them, and the factors that set their distribution are still not fully understood. With the exception of Ontario Lacus, the lakes on Titan occur in the northern hemisphere (these are shown in Figure 1) and 97% of the lakes on Titan are clustered in a region of 900 by 1800 km—about 2% of Titan’s surface area. The large lakes and small lacustrine depressions seen in Figure 1 can be roughly grouped into two principle modes based on size. The large lakes (several hundred kilometers in width) are thought to be deep (up to hundreds of meters in depth) [22,23,24]. They possess fractal shorelines and are connected to fluvial channels (e.g., Ligeia Mare, Figure 2) [25,26,27]. In contrast, the small lacustrine depressions appear to be more shallow depressions with rounded shorelines and show a gradient with elevation [28,29]. Empty depressions, which appear similar to the small lakes are found at elevations about 250 m above the similarly shaped small lakes (as discussed by Cornet *et al.* [6]). This could be indicative of the presence of aquifers and subsurface connectivity establishing defined liquid levels. [6,28,30].

The geological processes that form the lakes, both large and small, on Titan are still unclear. Recently Cornet *et al.* [6] reviewed the current state of knowledge of the lakes and proposed karst-like formation processes resulting from the dissolution of solid organics by the liquid methane and ethane.

**Figure 3:**
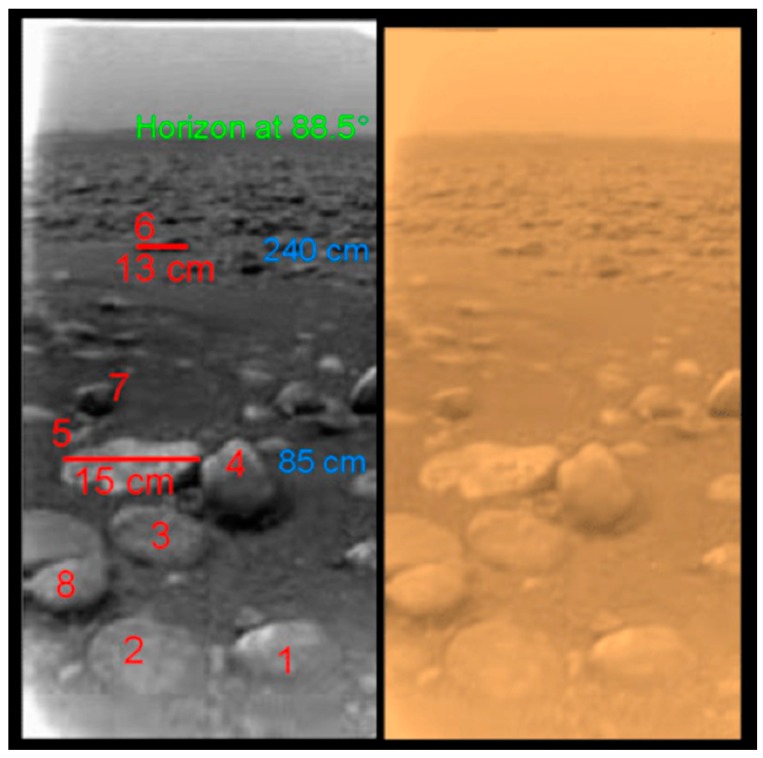
Titan’s surface at the Huygens landing site, 10.2°S, 192.4°W. There are at least eight rocks visible in the image—numbered in red with size indicated for two of them. Distance from the lander is shown in blue and the horizon at 88.5**°** is labeled in green. The small rocks are thought to be H_2_O ice mostly coated by organic solid material. The rounded nature of these rocks suggests past fluvial activity. The right panel shows, approximately, the true color of the scene. Direct measurements by the Huygens probe indicated that the ground at the landing was moist with methane and ethane at the time of the landing, 14 January 2005. Image from ESA.

Orbital spectral data have lead to the characterization of Titan’s surface into five terrain types: (1) bright terrain; (2) dark equatorial dune fields, or dark brown units; (3) blue units; (4) 5 μm bright units; and the (5) dark lakes [31,32]. The spectral data in the regions inside and around some polar (and equatorial) lacustrine depressions indicate the presence of various hydrocarbons and nitriles [33,34] and are not compatible with the presence of water ice exposed on the surface [35]. Rannou *et al.* [19] find that the surface spectrum at the Huygens landing site indicates a layer of water ice grains overlaid by a moist layer of weakly compacted haze particles. The spectrum is inconsistent with dry haze particles. Rannou *et al.* [19] could not locate the Huygens landing site, shown in Figure 3, on a specific type of terrain, because it is on the border between light and dark surface units. Extrapolating their spectral analysis of the Huygens landing to other equatorial regions, Rannou *et al.* [19] conclude that the ground at low latitudes is persistently wet, resulting either from a subsurface rich in liquid or from frequent enough rains to maintain moist surface conditions against evaporation. Williams *et al.* [36] suggested that moist surface conditions could persist for 5 to 50 days after a rain event, depending on moisture level. Rain in equatorial latitudes has been observed [37]. The CH_4_ and C_2_H_6_ liquids are widespread on Titan in lakes and in the moist surface. If life can exist in such liquids, life should be widespread on Titan.

### 2.4. Transport Cycles of Liquid Moving Nutrients and Wastes

On Earth, natural waters can be treated as a mixture of water and a non-volatile solid (salt). The amount of dissolved air in the water is negligible. Evaporation separates the volatile water from the salt-creating fresh water, defining in the process two somewhat different water habitats on Earth: freshwater—rivers and lakes, and salt water—oceans. The extensive surface area of water on Earth and the resulting evaporation ensures that there is an active hydrological cycle continually producing rain and snow.

As discussed above, rain has been observed in a few low-latitude regions [37]. In addition, there is extensive convective cloud activity in the summer polar regions [38,39] and at mid latitudes [40]. Global Circulation Models provide rough indications of the latitudinal and seasonal variations in rain and surface evaporation [41,42,43,44]. Typically, rain much in excess of evaporation is predicted at latitudes >60° higher than 60° during summer, in agreement with the presence of lakes, whereas middle to low latitudes experience net evaporation, in agreement with the absence of lakes—but still consistent with moist-soil conditions persisting following frequent rain.

In contrast, on Titan liquids are composed of three major components: methane, ethane, and dissolved atmospheric nitrogen. The solubility of N_2_ in the methane and ethane liquids is significant, up to 20% [45]. Ethane is non-volatile compared to methane and nitrogen and is left behind as the liquid evaporates. Nitrogen and methane (and perhaps ethane to a smaller extent) are present in the atmosphere and can equilibrate with surface liquids. Rain on Titan is expected to be a mixture of these gases [45] and the fraction of methane and ethane depending on the mixing ratio of ethane in the lower atmosphere, which is not known [36].

Tan *et al.* [46,47] and Luspay-Kuti *et al.* [48] have shown that the mixture of three components of differing volatility creates unusual behavior during evaporation and condensation, compared to a single component fluid. In particular, they find that the density of the fluid increases with temperature, and thus liquids in the polar regions are less dense than those near the equator, due to the compositional effects on density, exceeding those of thermal expansion. They also show that the liquid density decreases with pressure, very much unlike water on Earth. These properties affect the global circulation of fluids on Titan. Thus, the cycling of liquid on Titan, and its various compositions, is complex when compared to the cycling of water on Earth.

These variations in composition appear to be reflected on Titan. The composition of the northern lakes appears to be dominated by methane [48], whereas ethane has been identified as a major component in the southern-hemisphere Ontario Lacus [16,48], which may indicate that it is a remnant evaporitic basin [6] analogous to the Dead Sea on Earth. In contrast with Earth however, it is not clear that the liquid becomes less habitable as it is enriched in the lower volatile component. In fact, ethane is a better solvent than methane or nitrogen, and thus ethane-rich lakes may provide a richer more diverse solution for growth. For example, ethane is ~20× better than methane regarding solubility of small organic molecules [49].

Cornet *et al.* [6] suggest that if the lakes are karst features, then Titan’s lakes could be considered to be similar in age with some of the youngest features of the moon (less than ~100,000 year). In addition, karst formation rates in the northern mid-latitude (60**°**) regions should be more than three times faster than in the southern mid-latitude regions because in the present climate, GCM models predict that the southern latitudes receive less total precipitation, albeit with more intense rainstorms than northern latitudes. Cornet *et al.* [6] suggest this could explain why Titan’s south polar regions are devoid of well-developed lacustrine depressions, compared to the north.

The timescale of seasonal cycles is 30 times longer on Titan than on Earth just due to the orbital period. The diurnal period on Titan is 16 Earth days but nothing in the environment changes on the diurnal schedule. The CH_4_ and C_2_H_6_ liquids on Titan participate in an active hydrological cycle including rain, evaporation, lakes, and surface moisture. The cycles produce variations in composition of the liquids with latitude and seasons. Such cycles seem adequate for the transport of nutrients and wastes needed for life.

## 3. Carbon Biochemistry on Titan

Life on Earth is based on carbon biochemistry in the medium of liquid water. The postulated life on the surface of Titan is carbon biochemistry in a medium of liquid methane and ethane. Many of the key biochemical structures that life on Earth uses are specific to solvents with properties like liquid water. These include, for example, lipid bilayers as membrane components, amino acids and their collection into folded proteins, and the shape of DNA. Carbon biochemistry in liquid water on Earth is able to create the following: compartmentalization for autonomy and reproduction, information storage molecules and a way to duplicate them, structural molecules and a way to build them. For life on Titan similar structures must be created in a solution of methane and ethane.

### 3.1. Compartmentalization for Autonomy and Reproduction

It is generally thought that the ability to create a boundary layer between the interior of a living system and the external environment is a requirement for life [50,51]. On Earth, this is accomplished using a lipid bilayer, where the nature of the membrane is the result of the interaction of the bi-polar lipids with liquid water. Based on computational chemical analysis, Stevenson *et al.* [52] proposed a type of membrane which they termed “azotosome” capable of forming and functioning in liquid methane at cryogenic temperatures. The membrane is composed of small organic nitrogen compounds, such as acrylonitrile. The structural integrity of the membrane results from the attraction between polar heads of short-chain molecules that are rich in nitrogen and the interlocking nitrogen and hydrogen atoms that reinforce the structure. Although the azotosome structures may be difficult to construct in the laboratory, the structures provide a persuasive example of a possible membrane system for life on Titan.

### 3.2. Information Storage Molecules and a Way to Duplicate Them

Benner *et al.* [1] have observed that polymeric molecules suitable for information storage, such as DNA, must have the property that the shape of the molecule does not depend on the information stored in it. The shape of the biopolymer DNA is insensitive to the sequence encoded by that DNA. In contrast, the shape of a protein, another biopolymer, can be radically altered by the change of even a single amino acid in the sequence. Thus DNA is a suitable information-storage molecule and proteins are not. Following this approach, Benner’s research group [53] experimentally investigated the solubility of polyethers in non-polar hydrocarbon solvents at low temperatures. Such polyethers are a possible model for information-storage molecules in non-polar hydrocarbon solvents. While they find solubility in high-temperature hydrocarbons, they find that the solubility becomes immeasurably low below 170 K. McLendon *et al.* [53] note that overall, their results support a perspective elaborated by Bains [2] that water is a good solvent, primarily because it is liquid at high temperature, and secondarily because of its chemical properties (polar, high dielectric constant). In this view, the main difficulties with solution chemistry in Titan liquids are primarily the low temperature and secondarily the non-polar nature of the solvent. The search for a plausible information molecule for life in Titan liquids remains an open research topic. For the reasons discussed above, if such a Titan information molecule is discovered, it is likely that the binding of the information bits is achieved via hydrogen bonds. A possible approach for the molecular structure of the information system is a two-letter code involving hydrogen bonding with polar molecules containing O and N—both of which form hydrogen bonds and are available on Titan (O in H_2_O, and N in HCN, as well as in other nitriles). Another possible class of polymers that could store information is conducting polymers, such as polypyrrole and polyaniline, which are currently of much interest in the nanomaterials field [54]. These polymers are composed of C, N and H only and can transition between stable redox states—possibly a basis for information encoding.

### 3.3. Structural Molecules and a Way to Build Them

Life on Earth uses proteins as the primary structural molecule. Using ~20 amino acids to form proteins, life can construct a combinatorially large number of possible proteins. Due both to their internal interactions and especially to their interaction with water in both hydrophilic and hydrophobic ways, individual proteins fold into specific shapes. These shapes form the basis of structural molecules used by Earth life.

In Titan liquids, the structural substitutes for proteins might include hydrocarbon chains, aromatic ring structures, carbon nanostructures, including graphene, and various types of fullerenes. The addition of N to the carbon structures can potentially add diversity.

## 4. Ecology

On Earth, organisms, even microorganisms, live in communities. Within such communities organisms exchange matter and genes. Arguably, communities of microorganisms can survive and thrive in conditions that would be difficult for any of the individuals in the community to grow in in isolation. Communities can more efficiently cycle and recycle nutrients and can enhance genetic resilience. The liquid-water environment on Earth provides the medium for an organism to come into physical contact; as well, it mobilizes material released from cells. It worth noting here that viruses play a role in exchanging genetic information.

If life on Titan utilizes a set of biomolecules compatible with low-temperature methane and ethane liquids, then it is plausible that the same sort of ecological communication and exchange that occurs on Earth can occur in communities of Titan life forms. Signaling molecules could be low-molecular weight hydrocarbons that would be mobile in the Titan liquid. The role of NO and H_2_S in cell signaling in the human body shows that such signaling molecules need not be complex. If genetic material on Titan is based on soluble polymers as suggested by Stevenson *et al.* [52], then these also could be mobile in Titan liquid. Furthermore a Titan analog to viruses could be possible with hydrocarbon shells encasing raw Titan genetic polymers that attach and then insert the genetic polymers into host organisms. As discussed above, close recycling of elements may be essential for life on Titan, especially for metal atoms and H_2_O molecules, if these are used in catalysts.

## 5. Search

Given how different carbon-based life in liquid methane and ethane must be from carbon-based life in liquid water, it will be a challenge to implement a search strategy for life on Titan. However, some general principles that have emerged in the search for life may be applicable.

It has been suggested that a key feature of life is that it is selective in the basic molecules it uses [55,56]. Molecules with similar chemical properties can be present in biology in vastly different concentrations. Life on Titan would also have to select specific molecules from the possible variations. Thus, the prediction of McKay [56] should hold on Titan that a biological distribution of molecules would be a series of relatively sharp spikes while an abiotic distribution would be smooth. Surface material from Titan, for example the sediments at the shore of a lake, could provide a promising sample. An analysis of the nature of the molecules and their relative abundance could suggest a biological signal. This would be of great interest both as an indication of the presence of a biological system and as an inventory of the molecules used in the biochemistry of that system. One would know a great deal about life on Earth even if all that one knew were that life utilized the following: a certain twenty amino acids; a specific five nucleotide bases; and polysaccharides made up mostly from a few specific simple sugars.

The premier example of biochemical selectivity is chirality. This property is most well known in the amino acids used in proteins. Life on Earth uses only the L version of amino acids in proteins, not the mirror image, the D version. If life on Titan also uses molecules with chiral centers then detection of homochirality is a powerful indication of life. The simplest and most common chirality center is an atom that has four different groups bonded to it in such a manner that it has a non-superimposable mirror image. With N atoms added to hydrocarbons, chiral centers can be expected to form. Chirality can be determined by chromographic and optical methods, and thus may provide a robust and easily determinable method to test whether organic material in sediments on Titan are of biological origin.

### Ecological Change and H_2_

**Figure 4:**
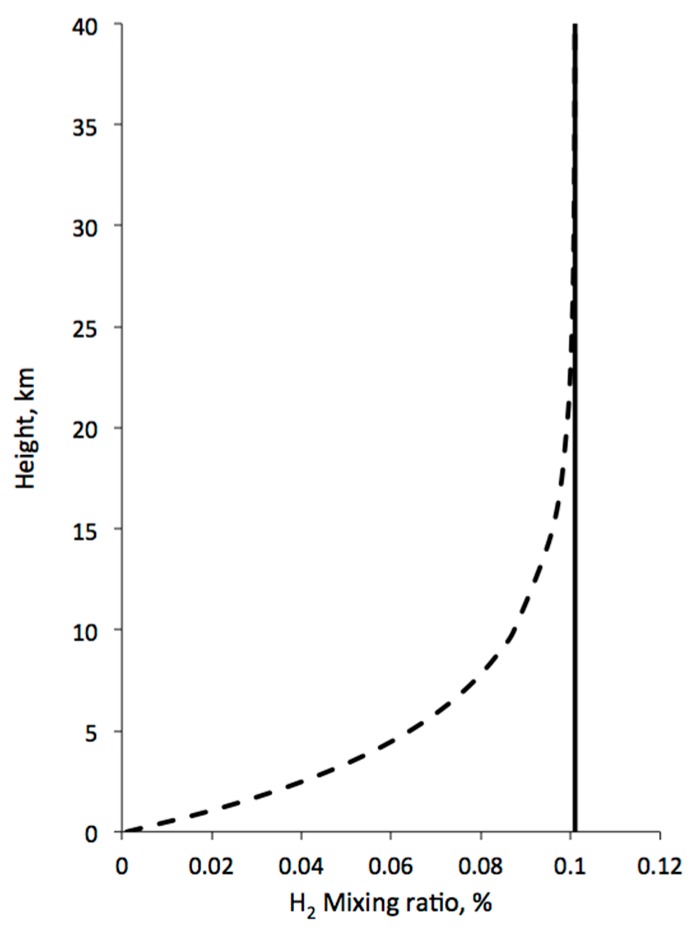
Schematic of expected H_2_ concentration in the lower atmosphere without a biological sink at the surface (solid line) and with a biological sink (dotted line).

Life that is widespread will effect the global environment. The majority of the O_2_, CO_2_, CH_4_, and even N_2_ in the Earth’s atmosphere are products of biology. The CO_2_ is of particular interest because its change with seasons reflects active growth. Local sources of CH_4_ also reflect biological processes. This approach may provide a method to search for life on Titan by analysis of the lower—possibly an easier task than collecting and analyzing lake-shore sediments. McKay and Smith (2005) suggested this approach and pointed out the H_2_ is the most promising atmospheric constituent on Titan to show a biological effect. If life is consuming atmospheric hydrogen, it will have a measurable effect on the hydrogen mixing ratio in the troposphere, if the biological consumption is greater than 10^9^ cm^−2^·s^−1^ [4]. Cornet *et al.* [6] review photochemical models for Titan and list production rates for C_2_H_2_, from 0.32 to 1.2 × 10^9^ cm^−2^·s^−1^ (e.g., [57,58]), and C_2_H_6_, from 1.2 to 15 × 10^9^ cm^−2^·s^−1^; thus if ~20% of the available C_2_H_2_ and C_2_H_6_ is consumed by methanogens, the corresponding H_2_ consumption should significantly deplete the near-surface H_2_ profile. This is shown schematically in Figure 4.

In addition to H_2_, McKay and Smith [4] suggested that methane-based life on Titan could consume acetylene and ethane. There seems to be evidence for depletion of acetylene and ethane on Titan. The data that suggest that there is less ethane on Titan than expected is well established [22]. Photochemical models have predicted that Titan should have a layer of ethane sufficient to cover the entire surface to a thickness of many meters but Cassini has found no such layer. Clark *et al.* [33] find a lack of acetylene on the surface despite its expected production in the atmosphere and subsequent deposition on the ground. There was also no evidence of acetylene in the gases released from the surface after the Huygens Probe landing [17,18]. Thus, the evidence for less ethane and less acetylene than expected seems clear.

The depletion of ethane and acetylene is more significant in the astrobiological sense, because of the report of a hydrogen flux into the surface. This is the key that suggests that these depletions may not be just due to a lack of production but are due to some kind of chemical reaction at the surface.

Strobel [59] predicts a strong flux of hydrogen into the surface. This determination is not the result of a direct observation but rather it is the result of a computer simulation designed to fit measurements of the hydrogen concentration in the lower and upper atmosphere in a self-consistent way. It is not presently clear from Strobel’s results how his conclusion of a hydrogen flux into the surface is dependent on the way the computer simulation is constructed or how accurately it simulates the Titan chemistry.

Courtin *et al.* [60] found that the latitudinal distribution of H_2_ appears to be non-uniform, with a mole fraction above 50°N larger than the globally-averaged value. This is unexpected, and Courtin *et al.* [60] speculate on an atmospheric process redistributing H_2_. One could also speculate that the ethane-enhanced southern hemisphere is biologically consuming more H_2_ than the methane-dominated northern hemisphere due to the superior properties of ethane as a solvent.

On Titan, the consumption of H_2_ and production of CH_4_ (Table 1) is obviously not a net source of CH_4_: it merely recycles CH_4_, thereby undoing the photolysis of CH_4_, and there is no *a priori* reason to expect the resulting CH_4_ to exhibit an isotopic shift from these reactions. The C-C bond in acetylene is strong, but this by itself does not imply a strong isotopic selectivity. For example, life on Earth breaks the strong bond between the N atoms in N_2_ without leaving a clear isotopic effect. Thus, the isotopic state of C on Titan is not definitive to the question of the presence of Titan methanogens. If there is an isotopic effect in the uptake of H_2_ this could be large, given the mass difference between D and H.

In overview, there are four possibilities for the recently reported findings regarding a flux of hydrogen into the surface of Titan. Here they are listed in order of their likely reality.

1. The determination that there is a strong flux of hydrogen into the surface may be mistaken. It will be interesting to see if other researchers, in trying to duplicate Strobel’s results, reach the same conclusion.

2. There could exist a physical process that is transporting H_2_ from the upper atmosphere into the lower atmosphere. One possibility is adsorption onto the solid organic atmospheric haze particles, which eventually fall to the ground. However, this would be a flux of H_2_, and not a net loss of H_2_.

3. If the loss of hydrogen at the surface is correct, the non-biological explanation requires that there should be some sort of surface catalyst, presently unknown, that can mediate the hydrogenation reaction at 95 K, the temperature of the Titan surface. That would be quite interesting and a startling find, although not as startling as the presence of life.

4. The depletion of hydrogen, acetylene, and ethane, could be due to a new type of liquid-methane-based life form, as predicted in [1,3,4].

Further measurements of hydrogen, as well as of acetylene and ethane abundances in the lower atmosphere of Titan, may be the most promising immediate strategy for a life search on that world.

Photosynthetic life might produce H_2_ and offset the biological sources. But the results would be seasonal changes in H_2_ by analogy with CO_2_ which is both produced and consumed on Earth but the relative rates vary with season (on Titan seasons are only changes in light levels).

In the context of role of H_2_ and life on Titan, it is interesting to note that proposals that energy derived from H_2_ reactions may have played a key role in the origin of life on Earth [61] may be modified and applied to the origin of life on Titan.

## 6. Summary and Conclusions

In this paper we have considered Titan as an abode of life based, of necessity, on aspects and the phenomenon of Earth and of life on Earth. The presence of a widespread liquid on its surface, the abundance of light and chemical energy, and the continual production of organic gases and solids all encourage the concept of indigenous life on Titan. Theoretical simulations have indicated a possible membrane system. However, laboratory investigations have failed to suggest a plausible information containing molecule. In sum, the question of Titan as an abode of life remains unresolved but indications so far warrant further investigation.

Based on what we do know, we can list the following fundamental challenges to life on Titan, in approximate order of severity.

1. The small diversity of elements available in the environment.
2. The low temperature of solution and the resulting negligible solubility.
3. The non-polar nature of the methane and ethane solvent, further lowering solubility.
4. The limited diversity of hydrocarbon structural molecules (compared to proteins).

Given these limitations, it may be that if there is life in the liquids on Titan’s surface it may be simple, heterotrophic, slow to metabolize, and slow to adapt with limited genetic and metabolic complexity. The simple molecules needed for metabolism may be widespread in the environment and in the methane/ethane liquids, but the complex organics needed for structural or genetic systems may be hard to obtain or synthesize. The communities formed may be ecologically simple—perhaps analogous to the microbial ecosystems found in extreme cold and dry environments on Earth.

The advantages of the Titan environment for life include: the free food from the sky in the form of organics (principally C_2_H_2_), the chemically benign nature of the non-polar solvent in terms of attacking biomolecules (in contrast to water), the lack of ultraviolet or ionizing radiation at the surface, and low rates of thermal decomposition due to the low temperature. Titan may have only a simple trophic system, probably without primary producers and without predators. Photosynthesis may be beyond the complexity that can be achieved with the limited element and hence genetic diversity—and food should be free. The simple low temperature life forms and communities envisaged would have very low energy demands and would grow slowly. Life on Titan may be not much more than auto-catalytic reactions encased in azotomes. However, if it had genetics, and were thus Darwinian, then what a wonderful life it would be: a second genesis different enough from Earth life to suggest that our Universe is full of diverse and wondrous life forms.

In terms of ethical considerations, the search for a second genesis of life on Titan contrasts with the similar search on Mars. On Mars, issues of contamination both with respect to the Martian environment and back contamination of the Earth are important concerns. Furthermore, it has been suggested that if there were a second genesis of life on Mars the global conditions are not favorable for it and humans may choose to intervene to improve them [62]. In contrast, possibilities of contamination of Titan with water-based life from Earth are essentially zero, as are the possibilities for back contamination of the Earth with life from Titan. In addition, if there is methane or ethane based life on Titan, the global environment is probably quite suitable for it, and it will not need human help for many billions of years.
